# Trade agreements and drug access: assessment of the impact of the 2009 Peruvian new drug policy on anti-infectives registration and availability

**DOI:** 10.1186/s40545-018-0151-0

**Published:** 2018-10-10

**Authors:** Lita Araujo, Michael Montagne

**Affiliations:** 1 Pharmaceutical Business and Administrative Sciences, MCPHS University, 179 Longwood Avenue, Boston, MA 02115 USA; 2 School of Pharmacy, MCPHS University, 179 Longwood Avenue, Boston, MA USA

## Abstract

**Background:**

The United States-Peru Free Trade Agreement required changes in the Peruvian pharmaceutical legislation that resulted in the National Drug Policy (NDP) of 2009. This study evaluated the registration of brand and generic anti-infectives before and after the agreement and implementation of the NDP and assessed the availability of anti-infectives in community pharmacies located in Arequipa-Peru.

**Methods:**

Anti-infectives registration database, provided by DIGEMID (Peruvian Drug Regulatory Authority), was evaluated from January 2005 to August 2014. Registration status included: new registrations, re-registrations, awaiting registration; or expired, denied, suspended, canceled and disregarded registrations. In addition, ten retail pharmacies located in different socio-economic areas in Arequipa were sampled in August 2014. Descriptive statistics and chi-square test were used for the analysis.

**Results:**

A total of 6112 anti-infectives registrations were categorized (5007 = antibacterials, 340 = antimycotics, 143 = antimycobacterials, and 622 = antiviral drugs). New registrations for brand and generic anti-infectives decreased from 2005 to 2013 (311 to 60 and 164 to 20 respectively). Re-registrations were from 121 (brand) and 115 (generics) in 2005 to 6 (brand) and 5 (generics) in 2013. Anti-infectives awaiting registration increased from 0 in 2005 to 351 (brand) and 137 (generics) in 2013.

The retail pharmacy survey included 1105 anti-infectives. These pharmacies carried 647 (58.6%) products awaiting registration, 74 (6.7%) expired (mostly combination of sulfonamides and trimethoprim followed by penicillin with extended spectrum, and fluoroquinolones), 4 (0.4%) suspended, and 2 (0.2%) denied registrations. Pharmacies in the low socio-economic area of the city had the highest proportion of generics (59.0% vs. 16.1%) from foreign origin (mainly India), and brand anti-infectives from Peruvian manufacturers (68.8% vs. 48.1%). High socio-economic areas had highest proportion of branded anti-infectives (83.9% vs. 41.0%).

**Conclusions:**

The new NDP reduced the number of brand and generic registrations; generics had the largest decline in registrations. Anti-infectives found in pharmacies located in low-income areas were more likely to be generics, and less likely to be currently registered by DIGEMID. The potential reduction in generic registrations resulting from the implementation of the NDP as a consequence of the bilateral trade agreement could result in lower availability of low cost medicines, but may increase the safety, efficacy and quality of marketed medicines.

## Background

Free Trade Agreements (FTA) are controversial for threatening important aspects of health especially access to affordable medicines. Multilateral, bilateral, and regional FTAs, as part of economic globalization, have included trade in health insurance, pharmaceuticals, and health services making health care reform no longer just a matter of national policy. FTAs make it difficult for countries to transfer from market-based health care systems to publicly funded health care programs once health care markets are opened to competition [1]. Many FTAs include provisions such as government procurement, competition policy, intellectual property (IP) rights protection, e-commerce, and more [2]. The IP rights about patent protection (explicitly the length of patent protection and second use patents) and data exclusivity are the provisions that could restrict the most access to generic medicines, and unfortunately, they have become the norm in the US [3, 4] and European trade agreements [5].

### FTA between Peru and the United States

On April 12, 2006, the United States of America and Peru signed the Trade Promotion Agreement. The FTA became effective on February 1, 2009; it aimed to improve the overall commercial and investment activity by eliminating or reducing tariffs on many goods including pharmaceuticals, accelerating the customs clearance process for US imports, and fortifying the protection of IP rights [6]. The IP chapter includes, among others, stronger protections for patents and test data as well as tough penalties for piracy and counterfeiting. The agreement restricted the grounds for invalidating patents and set up rules for protecting test data submitted for marketing approval of medicines (article 16.9 and article 16.10.2) [6].

Patent provisions were not changed, maintaining the 20 years of a patent’s life as with the World Trade Organization and the Andean Community agreements where Peru is a member. The amendment made to the FTA with respect to data exclusivity kept the 5 years of protection as it was proposed by the US (article 16.10.2(a)(b)) but added a modification: If the medicine is approved by the FDA (marketing registration) the term of protection starts running from the time of the first approval (article 16.10.2(c)(d)), thus reducing the protection period in Peru [6]. According to Rangel [7], this would provide better access to medicines while maintaining strong protection for innovation. However, according to Roca [8], Peruvian law does not require foreign companies to register first abroad, therefore they can register directly in Peru gaining the 5 years of data exclusivity.

### New drug policy and its connection to the FTA

The US-Peru FTA involved substantial changes in the Peruvian regulation to meet the requirements stipulated in the treaty. Law 29316 Amending, Incorporating and Regulating Miscellaneous Provisions on the Implementation of the Trade Promotion Agreement signed between Peru and the US of January 2009 sole purpose was initiating the FTA. The most important modifications related to the pharmaceutical sector were included in Article 5 that regulates data exclusivity, and Article 6 that replaced the requirements for the registration of pharmaceuticals, medical devices and sanitary products previously contained in Article 50 of the General Health Law 26842 of 1997 [9]. The General Health Law approved a simple procedure for the registration application process of pharmaceuticals that included an affidavit ensuring quality, safety and efficacy; analysis protocol from another country; and a free sale certificate. The procedure that originally lasted 15 days was reduced to 7 days (Table 1) [10].

**Table 1:** A comparison between the registration laws before and after the Free Trade Agreement presenting the main changes related to medicines

Articles	Law 26842 of 1997 and D.S. 010–97-SA [10]	Law 29459 of 2009 [14] and D.S. 016–2011-SA [13, 14]
Type of pharmaceutical product	- Brand medicines - Generic medicines - Diet products and sweeteners - Homeopathic products - Diagnostic agents - Biologic products - Radiopharmaceutical agents	- Medicines * Pharmaceutical specialties * Diagnostic agents * Radiopharmaceutical agents * Medicinal gases - Herbal medicines - Diet products and sweeteners - Biologic products - Compounding preparations
Requirements for registration and re-registration	- Affidavit assuring the quality, safety and efficacy of the product - Analysis protocol based on an authorized pharmacopeia of finished product - Free sale certificate and certificate^a^ of consumption (if product is imported)	- Application form with character of affidavit - Specifications and analytical techniques of APIs, excipients, final product - Validation of analytical techniques of finished product - Flow chart and validation of process of manufacture - Stability studies - GMP certificate granted by Digemid or from a country with HRS - Free sale certificate or certificate of pharmaceutical product^a^ (for import)
Timeframe application/evaluation process	Automatic with presentation of requirements, no more than 7 days	Between 45 days to 1 year according to the product’s category
Amount paid to get the registration license	10% of TU	59.74% of TU (category 1) 99.95% of TU (category 2) 99.65% of TU (category 3)
Term validity	5 years	5 years

^a^The Free Sale Certificate is an official document issued by the authority from the country of origin of the exported product that certified that the product is sold in the country of the manufacturer or exporter. The ‘certificate of pharmaceutical product’ from the International Commerce of WHO is a Free Sale Certificate

*APIs* Active pharmaceutical ingredients, *HRS* High regulatory surveillance, *TU* Taxation Unit

The Supreme Decree 001–2009-SA was issued to rule Law 29316, particularly the new requirements for the registration of pharmaceuticals, stating in part:

“The Trade Promotion Agreement …... establishes in its Chapter 16 provisions regarding the respect and safeguarding of Intellectual Property Rights, which must be incorporated into Peruvian legislation in this matter; …... its amendment in Law 29316, which establishes standards related to the protection of test data or other undisclosed data on pharmaceutical products, which must be regulated;

That, it is necessary to modify the system of registration of pharmaceutical products so that the health authority can demand certain information relevant to the evaluation and determination of the safety and efficacy of said products ……

This Supreme Decree shall enter into force on the date of entry into force of the Trade Promotion Agreement signed between Peru and the United States” [11].

The National Drug Policy (NDP) was approved on December 24, 2004 [12]. However, the NDP did not define a timeline for implementation and it was not initiated until the enactment of Law 29459 in 2009. The objectives of Law 29459 were to adapt the national drug regulation to the requirements of the FTA, to implement new drug registration requirements, and to reach the objectives of the NDP of universal access and rational use of medicines.

The Law of Pharmaceutical Products, Medical Devices and Sanitary Products 29459 of February 2009 and its supreme decree 016–2011-SA stipulated all regulations, new requirements and changes for such products (Table 1) and introduced the important terms of safety and efficacy within the regulatory authority and the Peruvian pharmaceutical sector. The Law stipulates the time allowed to review applications and grant marketing approvals according to the new categories of medicines:

- Category 1 (medicines in the essential medicines list): 45 to 60 days;
- Category 2 (medicines not in the essential medicines list but registered in countries of high regulatory surveillance (US, selected European countries, Japan, and Korea)): 45 to 90 days; and,
- Category 3 (other medicines): up to 12 months [13].

The fees for registration increased 10-fold and includes control activities and health surveillance. The technical requirements and the application documentation increased requiring presentation of therapeutic equivalence studies to demonstrate interchangeability, information on safety and efficacy (pre-clinical and clinical studies), a risk management plan for new medicines, Good Manufacturing Practice (GMP) certification, and analytical studies [14].

The technical information on safety and efficacy of the medicines must be submitted for registration and re-registration, but is not required for subsequent re-registrations unless it is required to address new safety and efficacy information. Registration and re-registration require studies of interchangeability; however, in vivo bioequivalent studies are only required for high risk medicines.

The Law also allowed from 3 to 10 years to comply with the requirements and studies for re-registration purposes. The GMP certificate must now be granted by DIGEMID, and quality control analyses are required for each lot that enters the market, except for biologics. Law 29,459 also includes chapters regarding universal access and rational use of medicines, promotion and research [14].

### Health care and pharmaceutical systems in Peru

The Peruvian health care system is divided into public and private sectors. The public sector comprises the Ministry of Health (MoH), the National Institute of Social Security (NISS), the health services of the Armed Forces and the National Police, the regional health boards, and the local government. The public health sector is financed by subsidies (indirect contributions) and by social security (direct contributions). The government manages and finances health services and medicines through Integrated Health Insurance with a low cost or no cost to people below the poverty or extreme poverty levels respectively. NISS provides free health care and medicines exclusively for salaried workers and their family members in their own hospitals and clinics. The private sector sells services to NISS in their clinics and doctor offices. The military and police have their own health system and infrastructure. The private health care system is mainly represented by clinics and other private entities like companies providing health insurance plans.

The MoH provides health services for 60% of the population; NISS provides health services for 30% of the population entitled to social security; and the Armed Forces, National Police, and the private sector together provide services to the remaining 10% [15].

The public sector’s procurement is both centralized for purchases and distribution of medicines at the national level, and decentralized for regional and local acquisitions. NISS performs centralized acquisitions of medicines and distributes them at a national level. Medicines are mostly distributed directly by the pharmaceutical manufacturers to hospitals and drugstore chains, also to large wholesalers, which mainly distribute brand imported medicines. Small wholesalers mainly distribute medicines to independent private community pharmacies. The retail sector has changed singularly; in the mid-1990s the market share of private pharmacies was around 86%, whereas in 2011 almost 60% of the market share was retained by drugstore chains [16].

The government’s universal insurance coverage and purchase strategies provide price regulation for medicines for the public sector. Whereas, the constitution protects free market competition and bans price control measures in the private sector.

### Study research questions

This study is intended to answer some of the research questions that arise as a result of the signing of the trade agreement and the subsequent implementation of the NDP.

- What is the impact of the NDP on the number of brand and generic anti-infectives registered in the country?
- What are the consequences of the NDP on the availability of anti-infectives at the retail pharmacy level?

## Methods

### Data sources

The evaluation was performed from January 2005 to April 2014 with the database provided by the Peruvian drug regulatory authority (DIGEMID).

The Anatomic Therapeutic Chemical (ATC) classification system at the WHO Collaborating Centre for Drug Statistics Methodology [17] was used to identify and categorize the anti-infectives from the DIGEMID database.

For the case study, data were collected from 10 retail pharmacies located in different socio-economic strata in the southern city of Arequipa, the second most industrialized and commercial city of Peru. Lima, Peru’s capital, was not chosen because the investigator wanted to determine the effect of the implementation far from the capital (in Peru, government policies are implemented first in the capital and then very slowly move to other parts of the country).

The metropolitan area of the province of Arequipa has 721 pharmacies and drugstores located among its 18 districts. Two pharmacies (1 privately owned by a university community health center, and 1 private with independent owner) were sampled from the low socioeconomic stratum representing 1.7% of the pharmacies in these districts. Two pharmacies (each from different pharmacy chains, one located inside a private clinic) were sampled in the districts of the high socio-economic stratum comprising a 1.4% sample. The other 6 pharmacies (3 privately owned, and 3 from different pharmacy chains) were representative of the middle socio-economic stratum comprising a 1.3% sample of the pharmacies in these districts. The socio-economic strata were determined using the poverty level per district from a study based on a population census of 2007 [18].

The districts with a poverty level of 26% or higher were considered in the low socio-economic stratum. The districts with a poverty level between 25 and 11% were considered within the middle socio-economic stratum and the districts with a poverty level of 10% or lower were categorized in the high socio-economic stratum [18]. The pharmacies were selected based on their location and type of pharmacy within the private sector. A convenience, non-random sample was selected from three different socio-economic strata representative of the city.

### Data manipulation

The impact of the regulations on the pharmaceutical market was estimated by creating a registration history for each anti-infective in the period of 2005 to 2014, before and after the implementation of both the FTA and the NDP. The registration history included 8 statuses: 1 = New registration; 2 = Re-registered; 3 = Awaiting registration; 4 = Expired; 5 = Canceled; 6 = Not approved; 7 = Deserted or Disregarded; 8 = Suspended.

For the case study, pharmacies were visited only once. The data were collected from July 30 to August 15, 2014. The following information was recorded: brand name, international nonproprietary name of anti-infective, registration number, dose and manufacturer.

### Data analysis

The frequency of anti-infectives registered for the first time was determined using the variable ‘authorization date of first registration.’ The number of anti-infectives re-registered in the study period was determined using the ‘authorization and expiration date’ of their registration considering that the license last 5 years. The anti-infectives for which companies filed applications for registration at DIGEMID but did not obtain the authorization and registration number yet were considered as ‘awaiting registration’. This situation started happening in 2008–2009 when the NDP was implemented, before the registration process lasted only 7 days. The number of anti-infectives awaiting registration was determined by the variable ‘status of application’ that was obtained from the DIGEMID database.

The DIGEMID website index was used to update the information from the database until August 2014 for statuses 3 to 8 (awaiting registration, expired, canceled, not approved, deserted or disregarded, and suspended registrations). The index is updated every month. The updated information was applied in the next part of the research, the case study.

### Case study

A case study was performed to determine the availability of anti-infectives at retail pharmacies comparing these with the anti-infectives registered through DIGEMID. Data from the 10 retail pharmacies were matched with the anti-infectives DIGEMID database from January 2005 to August 2014, to record the registration history of each anti-infective. The proportion of generic and brand anti-infectives sold in each retail pharmacy was also calculated as well as their country of origin.

The registration expiration date and the ATC pharmacological-chemical class were determined for products with statuses 4 to 8.

### Statistical analyses

Microsoft Office Excel 2013 was used to perform descriptive statistics. Sigma Plot 11.0 was used to perform chi-squared tests to assess differences in proportions. Mann-Whitney U test was performed when the chi-squared test determined statistically significant differences within the proportions. *P*-values < 0.05 were considered statistically significant.

## Results

A total of 6112 anti-infectives with a unique health registration number were extracted from the DIGEMID database (January 2005 to April 2014) using the ATC classification system. There were 5007 antibacterials; 340 antimycotics; 143 antimycobacterials; and 622 antivirals.

Table 2 shows the 8 types of registration statuses used in this study. The number of anti-infectives with new registrations was quite consistent from 2005 to 2009 with 475 and 448 registrations respectively; however, the number of new registrations decreased from 2010 to 2013, with 91 and 80 respectively (Table 2). The number of anti-infectives that were re-registered declined from 236 in 2005 to 11 in 2013. There was a statistically significant difference (*p* < 0.001) between the type of registrations before and after the legislation. There also was a statistically significant difference for new registrations (*p* = 0.016) and re-registrations (*p* = 0.032) before and after the implementation of the new legislation. The awaiting registrations started with 48 in 2009, increasing to 488 in 2013.

**Table 2:** Registration statuses of anti-infectives (n) from January 2005 to April 2014

#	Registration status	2005	2006	2007	2008	2009	2010	2011	2012	2013	2014^a^
1	New registrations	475	528	510	623	448	91	60	140	80	35
2	Re-registrations	236	228	225	257	181	195	138	68	11	0
3	Awaiting registration	0	0	0	0	48	66	146	256	488	190
4	Expired	466	301	349	352	212	355	406	364	365	249
5	Canceled	10	13	43	99	60	47	44	23	14	15
6	Not approved	0	0	1	12	24	36	13	11	0	0
7	Deserted/Disregarded	0	0	0	0	5	10	7	11	2	0
8	Suspended	0	0	0	1	0	2	2	2	0	5

^a^Through April

The number of registrations that expired from 2005 to 2013 went down and then up; in 2005 there were 466 expired registrations, in 2009 there were 212, and in 2013 there were 365. Furthermore, the canceled registrations increased from 10 in 2005 to 99 in 2008 and dropped again to 14 in 2013.

### New registrations

The proportions of brand anti-infectives new registrations were greater than the ones for generics throughout the study period, and this difference was even greater from 2009 to 2013. However, the number of new registrations gradually decreased from 2005 through the first four months of 2014 (Table 3). There was a statistically significant difference (*p* < 0.001) in the number of brand and generics new registrations before and after the legislation. Bivariate analysis also found a statistically significant difference in the proportion of brand (*p* = 0.016) and in the proportion of generics new registrations (*p* = 0.016) before and after the legislation.

**Table 3:** Number and proportion of new registrations of brand and generic anti-infectives from January 2005 to April 2014

Years	Brand		Generic		Total
	n	%	n	%	N
2005	311	65.5	164	34.5	475
2006	335	63.4	193	36.6	528
2007	332	65.1	178	34.9	510
2008	451	72.4	172	27.6	623
2009	364	81.3	84	18.8	448
2010	70	76.9	21	23.1	91
2011	45	75.0	15	25.0	60
2012	103	73.6	37	26.4	140
2013	60	75.0	20	25.0	80
2014^a^	26	74.3	9	25.7	35

^a^Through April

### Re-registrations

The proportions for brand and generics were quite similar from 2005 to 2007. In 2008 brand anti-infectives reached 60.3% and generics reached 39.7% followed by small fluctuations through 2013, although, the number of re-registrations declined gradually from 2005 to 2013 for both brand and generics (Table 4). No significant differences (*p* = 0.064) were observed in terms of number of brand and generic anti-infectives re-registered before and after the implementation of the new legislation. However, there was a statistically significant difference (*p* = 0.032) for brand anti-infectives re-registered before and after the implementation of the new legislation and for generic anti-infectives only (*p* = 0.016). There were no anti-infectives re-registered through the first four months of 2014.

**Table 4:** Number and proportion of brand and generic anti-infectives re-registered from 2005 to 2013

Years	Brand		Generic		Total
	n	%	n	%	N
2005	121	51.3	115	48.7	236
2006	116	50.9	112	49.1	228
2007	116	51.6	109	48.4	225
2008	155	60.3	102	39.7	257
2009	81	44.8	100	55.2	181
2010	114	58.5	81	41.5	195
2011	71	51.4	67	48.6	138
2012	32	47.1	36	52.9	68
2013	6	54.5	5	45.5	11

### Awaiting registration

The proportions for brand anti-infectives were 75.0% (*n* = 36) in 2009, 58.2% (*n* = 85) in 2011 and 61.1% (*n* = 116) by April 2014. In contrast, the proportions for generics were 25.0% (*n* = 12) in 2009, 41.8% (*n* = 61) in 2011 and 38.9% (*n* = 74) by April 2014. The awaiting registrations numbers gradually increased from 2009 to 2013 for both types of medicines.

### Case study

A total of 1105 anti-infectives were identified from ten community pharmacies in Arequipa, Peru.

**Fig. 1:**
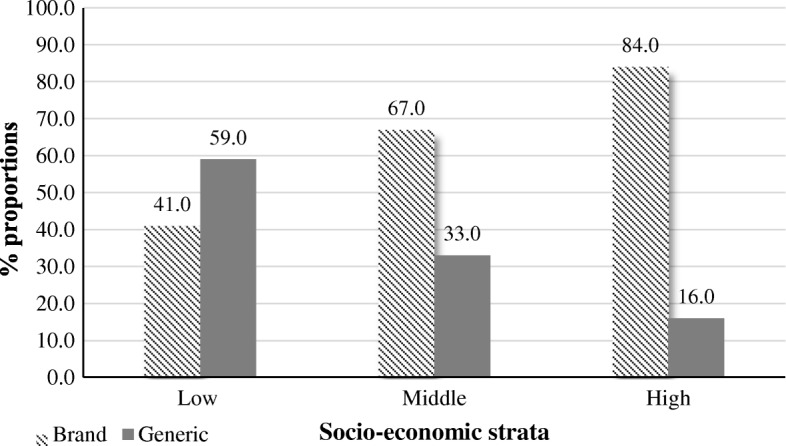
Brand and generic anti-infectives available at 10 retail pharmacies cluster by socio-economic strata

There were 59.0% of generics in the low socio-economic stratum pharmacies and 16.0% in the high socio-economic stratum pharmacies (Fig. 1). There was a relationship (*p* < 0.001) between brand and generic anti-infectives and their socio-economic strata.

**Fig. 2:**
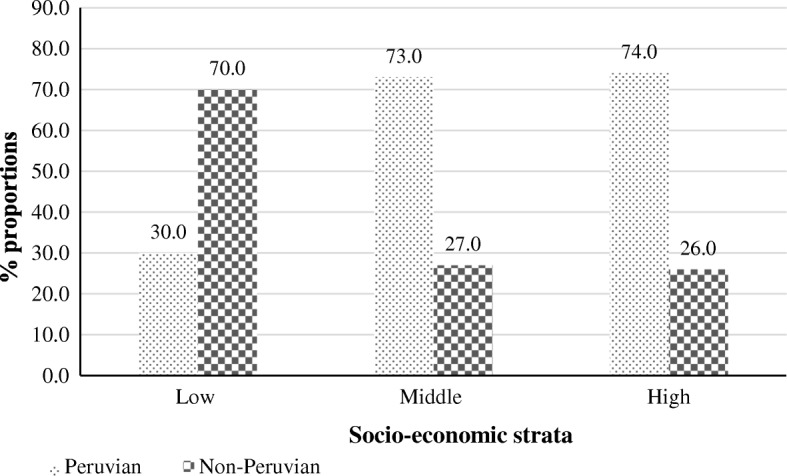
Peruvian and non-Peruvian generic anti-infectives available at 10 pharmacies in Arequipa-Peru divided by socio-economic strata

There were 69.0% brand anti-infectives of Peruvian origin in the low socioeconomic stratum and 68.0% and 48.0% in the middle and high socio-economic strata respectively. The non-Peruvian anti-infectives increased according to the socioeconomic stratum. In the case of generics (Fig. 2), the low socio-economic stratum pharmacies stocked 30.0% Peruvian anti-infectives and 70.0% non-Peruvian anti-infectives. However, the other two strata showed an opposite behavior: 73.0% and 74.0% Peruvian and 27.0% and 26.0% non-Peruvian anti-infectives.

The registration statuses of the anti-infectives found in the 10 retail pharmacies are shown in Table 5. The higher proportion of new registrations was observed in the pharmacies of the low socio-economic stratum; however, re-registrations were almost the same in the three strata as well as for those awaiting registration. Expired anti-infectives (status 4) were found in pharmacies in all three strata. The middle stratum pharmacies carried 2 anti-infectives with status ‘not approved’ and 2 with status ‘deserted/disregarded.’

**Table 5:** Registry statuses of anti-infectives by socio-economic strata from 10 retail pharmacies at Arequipa, Peru

#	Registration Status	Retail pharmacies by socio-economic strata					
		Low		Middle		High	
		n	%	n	%	n	%
1	New registrations	9	11.5	48	5.9	16	7.3
2	Re-registrations	22	28.2	228	28.2	53	24.3
3	Awaiting registration	42	53.8	471	58.2	134	61.5
4	Expired	4	5.1	56	6.9	14	6.4
5	Canceled	0	0	0	0	0	0
6	Not approved	0	0	2	0.2	0	0
7	Deserted/Disregarded	0	0	2	0.2	0	0
8	Suspended	1	1.3	2	0.2	1	0.5
	Total anti-infectives	78	99.9^a^	809	99.8^a^	218	100

^a^Percentage rounded error

There were 82 anti-infectives with statuses 4 to 8 found in the 10 retail pharmacies by August 2014. After eliminating duplicates and triplicates, there were 57 unique anti-infectives (Table 6). Of the anti-infectives with status 4 (expired registration), 43 were brand and 9 were generic products; and from these, 33 were of Peruvian origin and 19 of non-Peruvian origin. Also, 18 of these anti-infectives had a registration expiration date of 2014; 14 had a registration expiration date of 2013; and 9 had a registration expiration date of 2011. Furthermore, this table shows the ATC classification for anti-infectives with statuses 4 to 8 found in the retail pharmacies. The majority were the combination of sulfonamides and trimethoprim followed by penicillin with extended spectrum and penicillin with beta-lactamase inhibitors as well as fluoroquinolones.

**Table 6:** Characteristics of the 57 unique anti-infectives with statuses 4 to 8

Characteristics	Status 4 n	Status 6 n	Status 7 n	Status 8 n
Brand	43	1		1
Generic	9	1	1	1
Foreign	19		1	2
Peruvian	33	2		
Expiration date:				
2014	18			2
2013	14			
2012	5		1	
2011	9			
2010	3	1		
2008	1	1		
2007	2			
ATC – Anti-infective class:				
J01AA - Tetracycline	1	1		
J01CA - Penicillin extended spectrum^a^	9			1
J01 CE - Beta-lactamase sensitive penicillin	1		1	1
J01CF - Beta-lactamase resistant penicillin	1			
J01CR - Penicillin/beta-lactamase inhibitors	8			
J01DB - First generation cephalosporins^a^	3	1		
J01 DC - Second generation cephalosporins	1			
J01DD - Third generation cephalosporins	3			
J01EE - Combination sulfonamides/TMP^a^	11			
J01F - Macrolides, lincosamides	4			
J01G - Aminoglycoside antibacterial				1
J01MA - Fluoroquinolones^b^	8			
J01XD - Imidazole derivatives	1			
J02 AC - Triazole derivatives	1			

^a^Includes combinations with mucolytics/expectorants

^b^Includes combinations with phenazopyridine

*TMP* trimethoprim

## Discussion

The changes in the Peruvian drug legislation, as a consequence of the US-Peru FTA, have created uncertainty about their implications in the short and long term. Law 29459 caused considerable adjustments in the procedures for the registration of pharmaceutical products, medical devices and sanitary products. And the provision of data exclusivity may play a role in the long run.

### Impact of the NDP (law 29459)

The new law’s stricter requirements can explain the sudden decrease in the number of registrations. A study in March 2013, examined 11 procedures required by the MoH for the marketing of pharmaceuticals. The analysis of the perceptions of the companies found that the bureaucratic procedures were not consistent with the recent changes in the legal framework (Law 29459, and the DSs 014–2011-SA and 016–2011-SA); apparently increasing the time of DIGEMID’s procedures may affect the launch of new pharmaceuticals in the Peruvian market [19]. Furthermore, data provided by wholesalers estimated that each procedure can cost up to $13,433 per medicine. This cost did not include the opportunity cost of the time elapsed between the initiation of the procedure to obtain the marketing approval and the effective granting of the license. Although the registration fee and the cost associated with the new requirements can be considered modest by international standards, it might in fact become a market entry barrier for generic importers and domestic producers [16]. However, it can also contribute to the increase in the safety, efficacy and quality of registered medicines.

Since 2010, the government has requested GMP certificates in compliance with the new Peruvian standards. This requirement is also increasing the time needed to obtain marketing authorization. In 2011, Peru requested manufacturers, wholesalers, and importers of medicines to register and acquire the GMP from DIGEMID otherwise their medicines cannot be marketed in the country. DIGEMID has performed most of the inspections to production plants in China and India [20], countries with relatively low regulatory requirements.

The delay in approval of applications may also be related to logistical problems associated with the adaptation of DIGEMID reviewers to the new system that requires to evaluate pre-clinical and clinical studies. The previous system only required an affidavit proving safety, efficacy and quality.

The FTA required the Peruvian government to eliminate the 20% procurement subsidy for national companies. Now, national and foreigner companies can participate in public procurement under the same conditions. This will reduce the cost of medicines for the government but it could affect domestic pharmaceutical and/or wholesaler companies that would not be able to compete with a transnational company. This could be one of the reasons for the decline of Peruvian new registrations observed after 2009. Due to the pressures of the FTA, Peruvian companies could face intense competition but this can also reduce the presence of medicines without demonstrated safety, efficacy and quality.

Medicines in Peru are subjected to the following taxes: *Ad Valorem* import tax 9%, value-added tax (VAT) 16%, and local promotion tax 2%. However, medicines imported within the framework of the US-Peru FTA are exempted from the *Ad Valorem* import tax. Import duties applied to APIs and finished products are 6% and the VAT collected on finished products is 18% [21].

### FTA provision on data exclusivity

Data exclusivity is a requirement demanded by the US FTA (called TRIPS plus). Until a five-year period elapses, data exclusivity prevents DIGEMID from utilizing confidential trial data submitted by the originator company to demonstrate the efficacy and safety of generic drugs [6]. Cortés Gamba et al. [22] compared Colombia with Venezuela and Argentina, the last two countries do not have data exclusivity regulations, and concluded that the entry of generics depended on market considerations and data exclusivity had minor effect on market competition. On the other hand, two studies assessed the US-Jordan FTA and both concluded that the new data exclusivity regulation delayed generic entry and increased expenditures for medicines without generic competition [23, 24].

Data exclusivity has similar effect than a patent because it grants a temporary market monopoly and delays generic market competition. According to Seinfeld and La Serna [25], this practice would not affect generics competition because the generic companies typically wait between 3 to 5 years to see how the market for originators evolves before entering the market. However, data exclusivity can be especially important for medicines without patent protection or new formulations of existing medicines. In fact, in 2015, sponsor companies of 45 new chemical entities requested data exclusivity in Peru, of which 21 were enforced, 11 had already expired the exclusivity period, 11 were denied, and 2 were withdrawn [26]. As of October 2017, there were 27 new chemical entities including 6 antivirals with enforced data exclusivity and 5 new chemical entities including 2 antivirals waiting for first time marketing license that have requested data exclusivity [27].

### Anti-infectives availability at the retail level

The modest Peruvian pharmaceutical market concentrates its production on generics and branded generics of good demand. This is confirmed by the results obtained from the 10 retail pharmacies in which almost the double of brand and generic anti-infectives were of Peruvian origin. The higher proportion of Peruvian brand anti-infectives in the low and middle socioeconomic strata is an indication that these anti-infectives are branded generics, not originators. The slightly higher proportion of non-Peruvian over Peruvian brand anti-infectives in the high socio-economic stratum pharmacies are an indication of the consumption of originator anti-infectives in this stratum. One of the pharmacies from this stratum is located in a clinic where most of the patients have private insurance; therefore, the pharmacy mostly sells originator products.

Peru commercializes 3 types of medicines: originators, branded generics and generics; however, DIGEMID only categorize two types of medicines: brand name (that include originators and branded generics) and generics. Branded generics are products with the same active ingredient(s) as an originator but are permitted to differ in shape, size, labeling, and excipients. Branded generics are typically marketed using a brand name [28]. A study determined that from 80% of brand name products registered at DIGEMID in 2013 only 24% were originators, the rest were branded generics [26].

In Peru, generics and branded generics are typically pharmaceutically equivalent but not bioequivalent to the originator. Furthermore, in some cases, they are only pharmaceutical alternatives such as different salts, esters or complexes; or different dosage form or strengths but are prescribed or dispensed as interchangeable with the originator.

Peru’s new regulation requires studies of bioequivalence; currently enforced for high risk medicines. This could imply better acceptance of generics if the government informs the public about the improved quality of these medicines. Although, this requirement could increase generics prices and limit their access, independently of patents or data exclusivity, as an indirect effect of the FTA.

A study of the Brazilian pharmaceutical policy and access to essential medicines concluded that the goal of availability of essential medicines in the public sector has not been reached yet. However, the authors also mentioned that because of the regulations about quality tests, bioequivalence/bioavailability (mandatory for most generics and branded generics), and medicine registration (2 years registration time) there was an increase in the number of generics in the market compared to the small proportion of such medicines in the Brazilian market at the beginning of the generic’s policy implementation [29].

Indian and Chinese medicines are some of the cheapest in the Peruvian pharmaceutical market along with Latin American medicines. This explains the high percentage of generic anti-infectives of non-Peruvian origin in the low socio-economic stratum pharmacies. However, the middle and high socio-economic strata retail pharmacies kept a stock of Peruvian origin generics but from long time business manufacturers because they trust the quality of their products or because they are partners.

The sum of all 3 strata anti-infective stock reached 32.0% of generics and the rest were brand name. It is important to mention that 60.3% of generics are marketed in the public sector [30].

More than half of the anti-infectives in the 10 retail pharmacies were awaiting re-registration. Medicines applying for re-registration may continue in the market until the final decision is made. Anti-infectives awaiting registration can be anti-infectives that have not submitted GMP certificates, have failed plant inspections, or have not yet presented pre-clinical or clinical studies to prove safety and efficacy. Even more concerning, anti-infectives with expired registrations were found in all 3 pharmacy cohorts.

The anti-infectives mostly found were penicillins with extended spectrum, macrolides, cephalosporins, fluoroquinolones and combination of sulfonamides and trimethoprim (the government covers the treatments for HIV and tuberculosis in the public sector; this is why the majority of anti-infectives in the retail pharmacies were antibiotics). The public sector shows almost the same patterns, in a study conducted in four hospitals in four of Lima’s provinces, the antibiotics used with high incidence were amoxicillin, ciprofloxacin, metronidazole and azithromycin [31]. Unfortunately, the use of these antibiotics for common pathologies increase the risk of bacterial resistance. Moreover, the situation in the private sector is quite different since the retail pharmacies do not ask for a prescription, although officially required, and many of the antibiotics are sold for minor infections or no bacterial infections at all; in 2010 60% of anti-infectives were sold without a prescription in the private sector and 10% in the public sector [32].

### Limitations of the study

Only private settings were sampled for the case study; however, the study results have been compared and supported with public sector studies such as the Lima hospitals’ study. Furthermore, although growth in overall consumption of medicines is explained mainly by the public health sector, the total cost of such consumption is more related to consumption by the retail private sector [16].

The study included a small, convenience sample of pharmacies and the results of the study cannot be generalized to the situation in Peru. However, when the data were compared to DIGEMID reports, which are performed at the national level, the results did not differ greatly.

The present study could not measure directly if the availability of anti-infectives decreased after the FTA and NDP at the retail level. Although, the low proportion of new registrations in stock could mean that the availability of anti-infectives has been affected. Further studies could assess the effect of the FTA on the prices of medicines. It maybe still early to assess the impact of data exclusivity on the access to generics; a previous study concluded that 10 years are reasonable to measure the effect on prices and access of such medicines [22].

## Conclusions and Recommendations

This study found that the number of new registrations and re-registrations of anti-infectives dropped considerably after the implementation of the NDP in 2009. This drop is related to the longer time required for registration, the bioavailability and bioequivalence requirements, the GMP certificate requirement, and the first lot quality control defined by the NDP after the signing of the FTA.

The new regulation may affect the number of new registrations and the availability of affordable anti-infectives, and may also increase the safety, efficacy and quality of marketed medicines.

The following recommendation should be taken into consideration by the Peruvian government:

- FTAs may provide opportunities for changes in regulatory systems and improving the safety, efficacy and quality of medicines. Regulatory changes may occur without external changes, however, it may be difficult to reach a consensus with the pharmaceutical market stakeholders.
- Reducing the barriers to market competition, including IP regulation, should be one of the goals of the NDP. Negotiation of future FTAs should prioritize access to high quality affordable medicines.
- There is a need for implementing a comprehensive Generic Drug Policy, as part of the NDP, and inform the public how the new regulations will improve the safety, efficacy and quality of generics. It is important that prescribers and patients understand these changes and increase their trust on generics.
- Reinforce monitoring and surveillance of retail pharmacies to control the quality of drugs marketed in the country. Pharmacy surveillance must also assess the existence of the pharmacy license, the presence of the pharmacist, and the compliance with the prescription-only requirement for anti-infectives and other drugs.

## Abbreviations

APIs: Active pharmaceutical ingredients
ATC: Anatomic Therapeutic Chemical
DIGEMID: *(Dirección General de Medicamentos, Insumos y Drogas)*: Peruvian Drug Regulatory Authority
FTA: Free Trade Agreement
GMP: Good Manufacturing Practice
HIV: Human Immunodeficiency Virus
HRS: High Regulatory Surveillance
IP: Intellectual Property
MoH: Ministry of Health
NDP: National Drug Policy
NISS: National Institute of Social Security
TU: Taxation Unit
US: United States
VAT: Value-added Tax
